# The mystery behind membrane insertion: a review of the complement membrane attack complex

**DOI:** 10.1098/rstb.2016.0221

**Published:** 2017-06-19

**Authors:** Charles Bayly-Jones, Doryen Bubeck, Michelle A. Dunstone

**Affiliations:** 1 Department of Biochemistry and Molecular Biology, Biomedicine Discovery Institute, Monash University, Clayton Campus, Melbourne, Victoria 3800, Australia; 2 ARC Centre of Excellence in Advanced Molecular Imaging, Biomedicine Discovery Institute, Monash University, Clayton Campus, Melbourne, Victoria 3800, Australia; 3 Department of Life Sciences, Imperial College London, South Kensington Campus, London SW2 7AZ, UK

**Keywords:** membrane attack complex, MACPF, pore-forming protein, pore-forming toxins, cholesterol-dependent cytolysin, complement pathway

## Abstract

The membrane attack complex (MAC) is an important innate immune effector of the complement terminal pathway that forms cytotoxic pores on the surface of microbes. Despite many years of research, MAC structure and mechanism of action have remained elusive, relying heavily on modelling and inference from biochemical experiments. Recent advances in structural biology, specifically cryo-electron microscopy, have provided new insights into the molecular mechanism of MAC assembly. Its unique ‘split-washer’ shape, coupled with an irregular giant β-barrel architecture, enable an atypical mechanism of hole punching and represent a novel system for which to study pore formation. This review will introduce the complement terminal pathway that leads to formation of the MAC. Moreover, it will discuss how structures of the pore and component proteins underpin a mechanism for MAC function, modulation and inhibition.

This article is part of the themed issue ‘Membrane pores: from structure and assembly, to medicine and technology’.

## 1. Introduction

### (a) The role of the membrane attack complex

The complement system, composed of over 35 proteins found in the plasma or bound to host cells, forms an integral part of the early immune response [1]. Three major complement cascades, the classical, the alternative and the mannose-binding lectin pathways, can activate the terminal pathway, including the formation of the membrane attack complex (MAC).

MAC can form on and directly kill Gram-negative bacteria [2,3]. It is particularly important in combatting *Neisseria meningitidis*, with genetic deficiencies in MAC components leading to recurrent infections [4–6]. MAC pores can cause cell death by osmotic flux [7], and it has been postulated that the assembled pore may allow the passage of lysozymes across the outer membrane to degrade the peptidoglycan layer [8,9]. Although the translocation of lysozyme through the MAC is implied through *in vitro* experiments, this concept is also supported by the known role of a close homologue, perforin. In the case of perforin, there is translocation of a range of proteins including granzymes from cytotoxic T cell granules into the cytoplasm of target cells to induce apoptosis [10,11].

While MAC function has been studied predominantly in the context of Gram-negative bacteria, it has also been shown to assemble on the surface of parasites [12], Gram-positive bacteria [13] and unwanted assembly on host cells. In nucleated host cells, membrane disruption results in cell death by apoptosis [14–16] or by lysis if sufficient MAC is present [17]. Unlike erythrocytes, which are lysed by a single channel [18], nucleated cells can shed deposited pores in order to overcome the effects of the MAC [19,20]. In some circumstances, sublytic levels of MAC are found to be pro-survival, which may influence nearby cells during an inflammatory response [21]. MAC deposition is implicated in a number of signal transduction pathways [22], such as G-protein and PI3 K signalling, and has been associated with platelet activation in host cells [23–25].

### (b) Molecular assembly of the membrane attack complex

**Figure 1.**
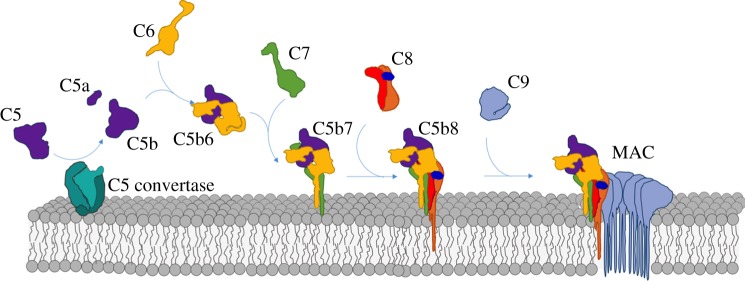
Illustration of the stepwise MAC assembly pathway from soluble complement factors. The first step requires cleavage of C5 (purple) into the small anaphylatoxin C5a and the large fragment C5b by the C5 convertase (turquoise). C6 (yellow) binds the labile C5b intermediate, resulting in a stable C5b6 complex. C7 (green) binds C5b6, anchoring the newly formed C5b7 complex to the membrane surface. C8, a heterotrimeric protein composed of C8α (orange), C8β (red) and C8γ (dark blue), is incorporated into the assembly precursor forming C5b8 and marking the first membrane penetrating event. Finally, multiple copies of C9 (light blue) join the assembly and span membrane, resulting in the final membrane attack complex (MAC).

MAC assembly commences with formation of the C5 convertase, a protease that triggers the sequential and irreversible trajectory along the complement terminal pathway (figure 1). C5 convertase cleaves C5 into two fragments: C5a and C5b. C5a is a potent anaphylatoxin that acts as a pro-inflammatory and chemotactic signal, promoting leucocyte activity and upregulation of immune responses [26–28]. C5b initiates MAC assembly on membranes in the immediate vicinity of activation. Similar to the transition of C3 to C3b, C5 cleavage results in dramatic conformational rearrangements within the C5b fragment [29–32]. Specifically, the C5b thioester domains (TED) and the ‘C1r/C1s, Uegf, Bmp1’ domains are released like a coiled spring and extend half-way down the macroglobulin scaffold. This exposes a transient intermediate that is captured by C6. C-terminal complement control protein (CCP) and factor I-like module (FIM) domains of C6, together with a short linker region, wrap around the extended TED stabilizing the interaction [29,33]. C7 binds the nascent C5b6 complex and the resulting C5b7 complex is lipophilic and is anchored to the bilayer independently of the convertase [34,35]. C8, a heterotrimeric complex comprising three polypeptide chains (C8α, C8β, C8γ), is incorporated into the membrane-bound assembly and undergoes a conformational rearrangement in which the C8α subunit becomes the first component to penetrate the lipid bilayer. The nascent C5b8 complex can then recruit a maximum of eighteen C9 molecules to form the final MAC pore [36].

### (c) General structure of membrane attack complex/perforin/cholesterol-dependent cytolysin proteins

**Figure 2.**
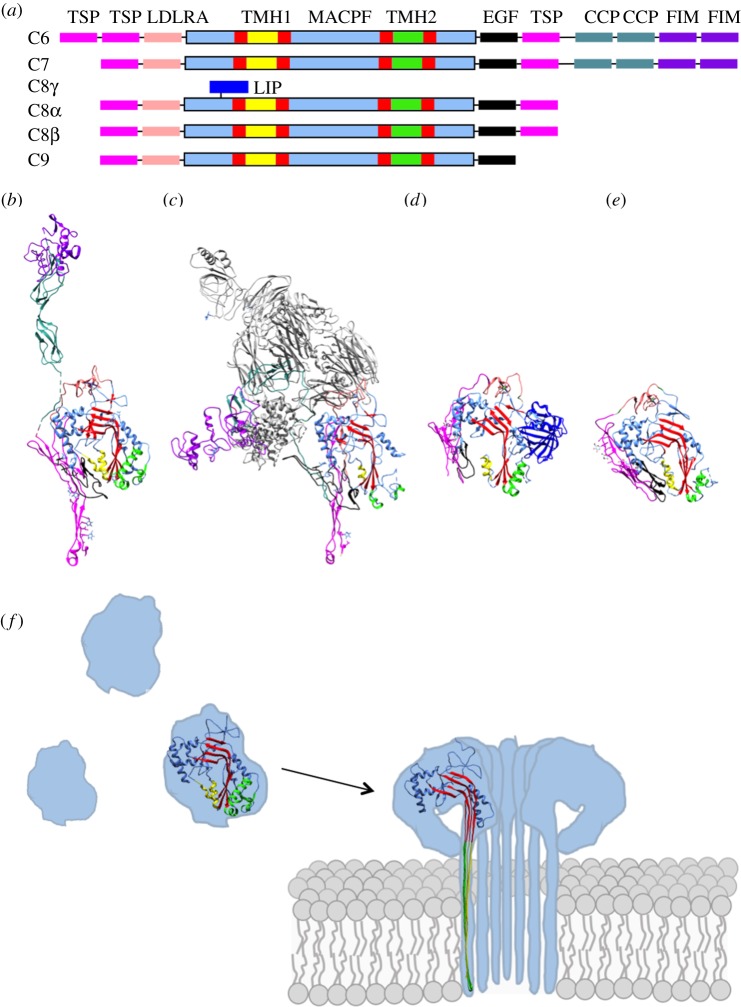
Domain architecture of complement MACPF/CDC-containing proteins; C6, C7, C8α–γ, C8β, C9. (*a*) Schematic showing domain organization. MACPF domain is coloured in a combination of blue, red, green and yellow, consistent with the colouring used in the structures shown in (*b*–*e*). Regions that form the final β-barrel pore are indicated as TMH1 and TMH2. The ancillary domains are as follows: thrombospondin (TSP) (magenta), low-density lipoprotein receptor type A (LDLRA) (light pink), lipocalin (LIP) (dark blue), epidermal growth factor type (EGF) (black), complement control protein (CCP) (teal), and factor I-like module (FIM) (purple). (*b*–*e*) Crystal structures of soluble MAC components. (*b*) C6 (PDB ID: 3T5O). (*c*) C5b6 (PDB ID: 4A5W), where C6 is coloured as in (*b*) and C5b is shown in grey. (*d*) C8αγ component of the C8 heterotrimer (PDB ID: 3OJY). (*e*) C8β component of the C8 heterotrimer (PDB ID: 3OJY). (*f*) The two TMH regions are shown as clusters of α-helices in the soluble monomer protein. Upon a dramatic conformational change, the TMH regions unfurl into β-sheets that span the target membrane. Colours are as for sections (*a*–*e*).

The sequence similarity between perforin and MAC components, C6, C7, C8α, C8β and C9, suggests a common domain responsible for membrane insertion. Structures of these proteins in their soluble forms defined the fold [29,33,37–39] (figure 2*a–e*), termed the membrane attack complex/perforin (MACPF) domain, and revealed an evolutionary link to the cholesterol-dependent cytolysin (CDC) family of bacterial toxins despite limited sequence identity [40,41]. As such, proteins across a wide range of genera and species that share this fold are often referred to as belonging to the MACPF/CDC superfamily [42]. The MACPF/CDC fold is composed of approximately 350 amino acids and includes a central antiparallel, twisted β-sheet. The central β-sheet is bent nearly 90° and is flanked by two clusters of α-helices. During conversion to the pore, both clusters unfurl to form two antiparallel transmembrane β-hairpins (TMHs) that comprise the final β-barrel [43–45] (figure 2*f*).

MACPF/CDC proteins form giant β-barrel pores that vary greatly in diameter. Despite the diversity across family members, all lesions are of a sufficient size to allow the passive and non-specific diffusion of folded proteins. The variable size of perforin pores leads to an inner pore of 13–18 nm while the MAC pores have an inner lumen of 11 nm [36,38]. In comparison, pleurotolysin forms smaller 8 nm diameter pores, while the most common suilysin pore size (37-mer) has a lumen of approximately 20 nm [46]. It is noteworthy that in model membranes, perforin and CDCs have also been observed to form incomplete rings [46–50]. These partial rings, referred to as ‘arcs’, can no longer recruit additional monomers due to their inserted state; however, they may still be sufficient in size to confer a lytic or transport activity [46,47]. While it is still unclear if MAC pores recruit additional C9 molecules after insertion, assembly precursors can penetrate the bilayer and demonstrate lytic activity [2].

### (d) General pore-forming mechanism for membrane attack complex/perforin/cholesterol-dependent cytolysin proteins

Based on structural and biophysical research on the CDCs, perforin, and the fungal toxin, pleurotolysin, the general mechanism whereby MACPF/CDC superfamily members form pores can be described in three key stages: membrane binding, oligomerization and membrane insertion [51,52] (figure 2*f*).

Membrane-binding of soluble proteins, through domains other than the MACPF/CDC domain, allows monomers to recognize and bind specific cell surfaces. CDCs interact with cholesterol in target membranes through their ancillary domain, ‘domain 4’. For a subclass of CDCs, which includes intermedilysin, species specificity is conferred through an additional interaction between domain 4 and the cell surface receptor CD59 [53,54]. Perforin binds the lipid bilayer through its C2 domain in a calcium-dependent manner [38]. The PlyB component of pleurotolysin docks onto a homodimer of PlyA, using an ancillary domain. The PlyA homodimer is responsible for specifically recognizing sphingomyelin and cholesterol-rich membranes [43]. In the case of CDCs, some research proposes that the membrane binding of its ancillary domain (domain 4) causes conformational changes in the MACPF/CDC and results in a heterotropic allosteric activation of surface-bound monomers [55]. However, this membrane-dependent allostery hypothesis remains to be structurally characterized and, furthermore, the observation of soluble forms of CDC oligomers contradict this hypothesis [56]. With respect to other members of the MACPF/CDC superfamily, there is currently no evidence for allosteric changes for either perforin or pleurotolysin. Furthermore, the presence of soluble polyC9 also supports that membrane-dependent allostery is not integral to the common MACPF/CDC assembly mechanism [57].

In the second stage, membrane-bound monomers laterally diffuse into an oligomeric prepore structure [58–60]. The flat shape of the MACPF/CDC domain comprises the dominant interaction interface, resulting in some of the largest oligomers characterized to date. Prepores can adopt both arc and ring geometries, with the stoichiometry of prepore rings ranging from an average of 13 for pleurotolysin to anywhere from 30 to 50 for CDCs [46,51,58].

In the third stage, dramatic conformational changes in protein structure enable insertion into the lipid bilayer. Conserved across all family members is the helix-to-hairpin transition of the TMH regions. Each monomer in the prepore unfurls two clusters of α-helices to form two β-hairpins, four β-strands in total. It is the association of amphipathic regions of these β-hairpins within the bilayer that give rise to the characteristic giant β-barrel pore architecture [61] (figure 2*f*). While research on pleurotolysin suggests a simultaneous insertion of β-hairpins in a zippering down trajectory [43], it remains to be seen if all MACPF/CDC-containing proteins undergo a simultaneous prepore-to-pore transition.

### (e) Membrane attack complex deviates from the canonical membrane attack complex/perforin/cholesterol-dependent cytolysin pore-forming mechanism

Unlike other MACPF/CDC domain-containing pores, MAC is a hetero-oligomeric complex and, as such, challenges the general three-step mechanism of the superfamily. There is no identified receptor or specific lipid dependency for the initial membrane-binding step. Pores can form on a variety of surfaces ranging from LPS envelopes of Gram-negative bacteria to liposomes. Structures of complement proteins reveal a highly conserved MACPF/CDC domain; however, ancillary domains are not homologous to the membrane-binding domains of perforin or CDCs. Structures of C6 and C8 show that the common ancillary domains for C9: thrombospondin (TSP), low-density lipoprotein receptor A, (LDLRA) and C-terminal epidermal growth factor (EGF)-like domains, are not in the equivalent position to the membrane-binding ancillary domains of other MACPF/CDC-containing proteins (figure 2). In contrast to perforin, CDCs and pleurotolysin, which bind membranes as monomers, MAC membrane-binding begins upon incorporation of C7 to the C5b6 complex. Furthermore, neither C8 nor C9 can interact with the membrane unless integrated into an already associated assembly precursor (i.e. C5b7 or C5b8, respectively).

Interestingly, MAC ancillary domains play an important role in the oligomerization of the complex rather than membrane binding. Similar to other well-studied pores [43,46], the MACPF/CDC domain is the major contributor to the oligomer interface [36,57]; however, the MAC's highly conserved N-terminal TSP1 domains contribute approximately a quarter of the buried surface area [57]. Indeed, deletion of C8α's N terminal TSP1 and LDLRA domains impacts MAC formation [62,63].

While all MACPF/CDC-containing proteins undergo a similar dramatic change in secondary structure of TMH regions upon pore formation, complement proteins do not undergo a vertical collapse towards the membrane. Similar to perforin and pleurotolysin, but distinct from CDCs [38,43], the C8 and C9 components of the MAC are predicted to have TMH regions that are sufficiently long to transverse the target membrane [41]. However, not all MAC proteins transverse the lipid bilayer. Indeed, C6 TMH regions are predicted to be too short to span the membrane [37]. Incomplete penetration is further supported by photolabelling experiments [64] and recent MAC structures, which reveal an irregular β-barrel pore [36,65] (discussed in §2). While the trigger for membrane insertion remains unknown, the conserved helix-turn-helix (HTH) motif of the MACPF/CDC domain, also referred to as CH3, is postulated to be involved. Comparison of the HTH region of monomeric C6 [37] and oligomerized C9 [57] shows a shift in its position and suggests a role in the release of the TMH2 region during pore formation.

## 2. Recent structures of membrane attack complexes

**Figure 3.**
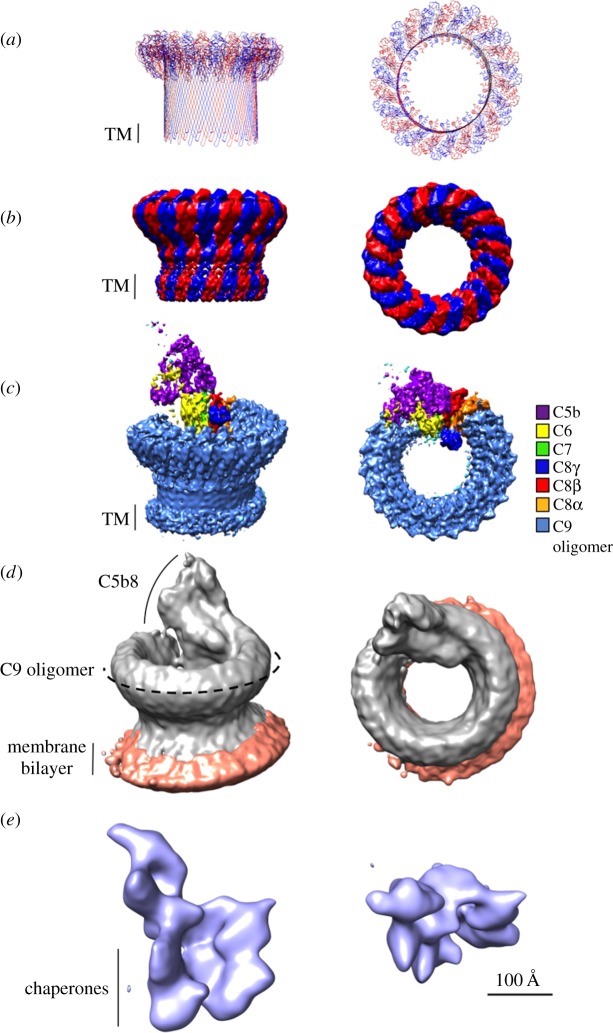
Comparable side and top views of polyC9, MAC and SC5b9 complexes determined using cryoelectron microscopy (cryo-EM). Pseudo-atomic model of a 22-fold symmetric C9 oligomer (PDB: 5FMW) (*a*) based on the polyC9 reconstruction (*b*) (EMBD: 3235). Alternating C9 monomers are coloured red and blue. Putative transmembrane regions (TM) are indicated by a black bar. (*c*) Cryo-EM reconstruction of the MAC pore (EMBD: 3134), where density for each protein component is coloured (see key). (*d*) Subtomogram average of the MAC on liposomes. Protein density is grey and lipid bilayer is orange (EMBD: 3289). (*e*) Soluble, regulated form of the MAC, SC5b9 (EMBD: 1991), in which non-MAC density is indicated by chaperones. Scale bar, 100 Å, is relevant for all structures.

While previous studies using negative stain electron microscopy [66–68] have provided the overall shape of the MAC, the molecular architecture of how different complement components come together has only recently been discovered [29,36,57,65] (table 1 and figure 3).

**Table 1.** Available structures of monomeric MAC components, intermediates and the final pore. Structures of an *in vitro* polymerized C9 (PolyC9), a soluble regulated MAC (SC5b9) and the MAC inhibitor CD59 are also listed. Cryo-EM refers to single-particle cryo-electron microscopy. Cryo-ET indicates a subtomogram average from a cryo-electron tomography reconstruction. NMR stands for nuclear magnetic resonance spectroscopy.

protein	technique	solution phase	resolution (Å)	year	accession no.	reference
C5	crystallography	soluble	3.1	2008	PDB: 3CU7	[69]
C5b6	crystallography	soluble	3.5	2012	PDB: 4A5W	[29]
C6	crystallography	soluble	2.9	2012	PDB: 3T5O	[37]
C8αγ	crystallography	soluble	2.1	2008	PDB: 2RD7	[70]
C8γ	crystallography	soluble	2.0	2007	PDB: 2OVE	[71]
C8αβγ	crystallography	soluble	2.5	2011	PDB: 3OJY	[39]
PolyC9	cryo-EM	soluble	6.7	2015	EMDB: 3235	[57]
MAC	cryo-EM	extracted from liposomes and detergent solubilized	8.5	2016	EMDB: 3134	[36]
MAC	cryo-ET	liposome embedded	23	2016	EMDB: 3289	[65]
SC5b9	cryo-EM	soluble	24	2012	EMBD: 1991	[29]
CD59	NMR	soluble	—	1994	PDB: 1CDQ	[72]
	crystallography	soluble	1.8	2007	PDB: 2UX2	[73]

### (a) Overall shape and function

Structures of the MAC reveal that the complex comprises a hollow cylindrical density with a single stalk protrusion. Similar to CDC and pleurotolysin structures [43,61], MAC's cylindrical shape [36,65] is consistent with a giant β-barrel pore in which each complement protein contributes two β-hairpins. Indeed, single cysteine labelling studies of C8 and C9 identified that the common MACPF/CDC domain contributed at least one transmembrane region per protein in the final pore [74]. This research disproves the hypothesis that MAC spans the target membrane using amphipathic α-helices, as postulated prior to structural information [75,76]. Images of MAC on liposomes [65,68] reveal a single transmembrane region at the base of the barrel, indicating the complex likely spans one bilayer; in the case of Gram-negative bacteria, only the outer membrane would be perforated by a single MAC. Finally, MAC structures show that the C5b8 component forms an asymmetric, monotopic complex that protrudes from the extracellular surface of the target membrane [36,65], in line with the previous structural studies of a soluble regulated form of MAC, SC5b9 [29] (figure 3*c*–*e*).

### (b) Split washer shape

One of the most striking features of the MAC pore is that it is an irregular β-barrel with a ‘split-washer’ configuration [36,65]. Asymmetric assembly precursors comprise an integral part of the pore, yet do not span the length of the bilayer [36,65]. TMH regions of assembly precursors are shorter than those of C9 and distort the lipid bilayer [36,65]. Differences between the symmetric and asymmetric regions of MAC prevent closure through a canonical MACPF–MACPF domain interface and contribute to its ‘split-washer’ shape. Moreover, the final MAC has a twisted β-barrel, which likely impacts biophysical properties of the local lipid environment and may play a role in MAC function.

### (c) Heterogeneity of the final membrane attack complex shape

Both MAC and CDC pores have been observed in a range of stoichiometries. Atomic force microscopy and electron microscopy analysis of CDCs and perforin reveal a variety of oligomeric states (see §1d). Furthermore, the recent cryo-ET study of the MAC demonstrated heterogeneous single pores as well as incomplete pores that join to make multimeric complexes [46,65]. By contrast, the single particle reconstruction of the MAC reported a largely homogeneous stoichiometry [36]. While this may reflect sample purification, both biochemical and *in silico* differences in lipid composition between the two studies may also play a role.

## 3. *In vivo* function

MAC can rupture cell membranes with a wide variety of lipid compositions. It lyses Gram-negative as well as host cells if not properly controlled. Therefore, another important aspect of understanding MAC assembly is to understand when the pore is not formed, i.e. when MAC formation is inhibited. A fine balance of regulatory factors on host cells quenches early complement activation and amplification to prevent the initiation of the MAC, thereby protecting the host cell from the immune attack [77]. However, once MAC assembly is initiated, other factors can still block formation of the final pore.

### (a) Inhibiting off-target membrane attack complex assembly

Activated complement components can assemble soluble, off-pathway products incapable of membrane binding. The plasma factors, vitronectin and clusterin, scavenge dead-end assemblies (referred to as SC5b7, SC5b8 and SC5b9) and prevent further oligomerization. In addition, C8 binding of non-membrane-associated C5b7 is one of the most potent inhibitors of MAC, preventing subsequent interaction with the lipid bilayer [78,79].

Assembly precursors initiated on host membranes can also be blocked from forming the pore by CD59 [80,81], a glycosylphosphatidylinositol (GPI) anchored protein that can bind to either C8 or C9. CD59 cannot interact with the soluble, plasma forms of either of these two components; therefore, it is postulated that the binding site is only revealed upon MAC assembly. Epitope mapping and mutational studies have identified residues on C8 and C9 that are important for binding [82–84]. Intriguingly, these amino acids also correspond to predicted transmembrane segments. Although no structural information exists for CD59-bound complement complexes, the crystal structure of CD59 in complex with a bacterial toxin that competes with MAC binding revealed a binding site that comprised a β-hairpin extending the central β-sheet of CD59 [53]. These data suggest that CD59 may recognize the β-hairpin structural motif of complement proteins whose α-helices have unfurled and trap it in a state unable to penetrate the bilayer.

Changes in the effective presentation of CD59 on a cell surface have profound biomedical consequences. Mutation in a gene involved in GPI anchor synthesis (*PIG-1*) prevents trafficking and presentation of both decay accelerating factor and CD59 to the host plasma membrane and leads to paroxysmal nocturnal haemoglobinuria [85,86], a disorder associated with unregulated MAC activity [87]. Conversely, increased presentation of CD59 on leukaemic cancer cells reduces the effectiveness of immunotherapeutic rituximab, which acts in part by activating the terminal pathway [88,89].

### (b) Membrane attack complex inhibition by pathogens

Pathogens have evolved a number of methods to evade killing by MAC [90,91]. Enveloped viruses, such as the HIV-1, can protect themselves by incorporating CD59 originating from the previous infected host's membrane [92]. Additionally, *Yersinia pestis* has been found to recruit vitronectin which in turn can inhibit MAC using an outer membrane protein called Ail [93]. Some pathogens express CD59 mimetics such as herpes virus saimiri that encodes HVS-15 [94,95] and SCIP-1 found on the platyhelminth, *Schistosoma mansoni* [96]. These adaptations form the basis for novel therapeutic targets and reflect a continuing evolutionary battle between hosts and pathogens.

## 4. Concluding statement

In summary, recent structures of the MAC have provided important mechanistic insight into MAC assembly and mechanism of action. The structure adopts a unique ‘split-washer’ and irregular β-barrel architecture, in which MAC precursors form an integral component of the pore [36,65]. Assembly is propagated by a combination of MACPF/CDC and ancillary domain interfaces, where differences in complement proteins result in a non-canonical MACPF/CDC closure. Nevertheless, higher resolution structures of MAC, in which TMHs can be visualized, will be necessary to define precise structural transitions that govern initial insertion and pore closure. Partial insertion of assembly precursors and the MAC's irregular twisted β-barrel architecture have raised new questions regarding its mechanism of action [36,65]. The importance of membrane distortion in both lytic and sublytic roles of MAC creates an exciting new area for future investigation.
